# Epidemiology of Cancer in the Fiji Islands: 2010 to 2018

**DOI:** 10.1177/10105395241306488

**Published:** 2025-01-28

**Authors:** Carol Kartika Naidu, Nicola Wiseman, Devina Nand, Neil Harris

**Affiliations:** 1 Griffith University, Gold Coast, QLD, Australia; 2 Ministry of Health & Medical Services—Fiji, Suva, Fiji

**Keywords:** Fiji, cancer epidemiology, public health, early detection, breast cancer, cervical cancer

## Abstract

Cancer is a global public health concern with increasing incidence and mortality rates, particularly in low- and middle-income countries (LMICs) like the Pacific Island Countries and Territories (PICTs). Among the PICTs, Fiji faces a growing burden of cancer. This study aimed to analyze cancer incidence and mortality data in Fiji from 2010 to 2018 to identify trends and provide an update on the current cancer-related statistics in the Fiji Islands. The top three cancer incidence rates among women were breast, cervical, and endometrial cancer, whereas prostate, liver, and lung cancer were the most prevalent among men. Notably, the central division had higher cancer incidence rates, whereas the northern division had a disproportionately higher mortality rate. Factors contributing to these trends may include lifestyle behaviors, limited access to health care in certain regions, and low awareness. Although this study has limitations due to data quality, it emphasizes the need for targeted interventions, accurate data reporting, and improved cancer treatment delivery to reduce the burden of cancer in Fiji.

## What we already know

- The burden of cancer is high in the Pacific Island Countries and Territories (PICTs), particularly in Fiji.
- Currently, there is a lack of comprehensive data on cancer mortality and incidence rates in Fiji.

## What this article adds

- The most common cancers among women were breast, cervical, and endometrial cancer, while prostate, liver, and lung cancer were the most prevalent among men.
- Mortality rates were higher in the Northern Division of Fiji, which may be due to factors such as lack of access to health care.
- There are cancer data quality issues, and there is a need for robust measures for accurate data reporting.

## Introduction

Cancer is a significant global public health issue contributing to one of the highest mortality rates when compared with other non-communicable diseases (NCDs). According to the World Health Organization’s (WHO) International Agency for Research on Cancer (IARC), the global cancer burden has increased to 19.3 million new cases and 10 million cancer deaths in 2020.^1^ The predicted global cancer burden by 2040 is more than 27 million new cancer cases per year, nearly 50% increase over the estimated cancer cases in 2018, with most cases occurring in low- and middle-income countries (LMICs). Although statistics show that cancer mortality rates are decreasing in many high-income countries (HICs), they are increasing in LMICs, particularly in countries in the in the Pacific.^2,3^

Nearby countries such as Samoa have very high incidence rates of cancers, such as breast and prostate cancer, with age-standardized incidence rates of 88.8 per 100 000 and 49.1 per 100 000, respectively. In most Pacific Island Countries and Territories (PICTs), cancer surveillance systems are not well developed, and some patients tend to present late with advanced-stage cancers. Many PICTs are unable to provide cancer care to those affected, with some patients not receiving care at all, receiving restricted treatment only, or being treated abroad.^4^ Treatment abroad poses a substantial economic burden on the patient, their family, and on the country if the treatment is government funded; however, in many instances, this is the only available option, as many PICTs do not have any, or have restricted access to, cancer screening, pathology, oncology, surgical, and palliation services.^5^ Unsurprisingly, this will become a complex public health challenge in countries such as the Fiji Islands, which has one of the highest prevalence of cancer in the Pacific Region, with the incidence of cancer projected to drastically increase in the next 20 years.^6^

Fiji’s health system is sustained by the public health sector and government-funded health facilities. Fiji consists of two main islands, Viti Levu and Vanua Levu, and is divided into four divisions: the Central, Western, Eastern, and Northern. There are three main hospitals, one in each of the Central, Western, and Northern Divisions, that are able to offer cancer diagnostic and treatment services. The Eastern Division is more isolated, and all health services there are redirected to the Central Division.^7^ Cancer contributes significantly to the burden of illness experienced by both women and men in Fiji.^8^

Cancer is the third leading cause of death in Fiji, and the number of new cases is rapidly increasing. In Fiji, the risk of developing cancer before the age of 75 is 17.2%, whereas the risk of dying from cancer before the age of 75 is 10.1%.^9^ Breast cancer was the leading cause of death and incidence among women in Fiji, with the number of cases expected to increase by 29.5% over the next two decades.^9^ Prostate cancer, on the contrary, accounted for nearly 33.3% of all cancer cases in men, with a mortality age-standardized rate (ASR) of 12.9 per 100 000 people.^10^ Social determinants have a significant impact on cancer incidence rates and cancer outcomes within the Fiji Islands; for example, populations with lower socioeconomic status and living in rural areas typically have poorer health outcomes.^11^ Therefore, it is crucial to investigate the specific barriers hindering the uptake of cancer-related services and understand the factors that contribute to delayed detection and treatment.

To address these concerns, this study utilized secondary data provided by the Data Analysis and Management Unit (DAMU) in Fiji to analyze the most recent data on cancer incidence and mortality in Fiji from 2010 to 2018. In addition, it will examine the burden of incidence and mortality in different geographical regions within Fiji and the top three sites for incidence and mortality among both males and females. The analysis aimed to establish a foundation for future research by uncovering barriers to cancer health care uptake, leading to a better understanding of the factors hindering the utilization of cancer-related services. These insights will inform strategies to promote early detection and timely treatment. To the best of our knowledge, this is the first study that analyzes and presents comparative data for both males and females and multiple cancer types in the Fiji Islands.

## Methods

The DAMU is a division of the Ministry of Health and Medical Services (MHMS) in Fiji. Its responsibilities include the collection, verification, and storage of all data pertaining to the nation’s health care system. In addition, the DAMU offers training as well as assistance in the areas of data collection, data analysis, and the utilization of information to users of both manual and electronic health information systems. The vast majority of information regarding cancer is gathered by the pathology departments located within the three primary divisional hospitals. Pathology reports, Medical Cause of Death Certificates (MCDC), and PATISPlus are the primary resources that the DAMU uses to obtain data pertaining to cancer. PATISPlus is the hospital data management system where most hospital records are entered digitally.

The DAMU employs Microsoft Access as a tool to record and compile cancer data from various sources, including pathology reports, PATISPlus, and the MCDC. Instead of a dedicated cancer registry, the DAMU database serves as a comprehensive repository for cancer-related information. However, it is crucial to emphasize that Fijian regulations impose limitations on the disclosure and publication of specific data, such as ethnicity information. Consequently, this study is unable to analyze and compare cancer incidence and mortality rates across different ethnic populations due to these regulatory restrictions.

The data set for this study was comprised of cancer surveillance (incidence and mortality) data reported to the DAMU between 2010 and 2018. To assign codes to cancer histology in the cancer-related database, the WHO International Classification of Diseases for Oncology (ICD-O) was used. These codes were assigned based on how pathology laboratory reports described the histology. The data extracted include the following categories of information: age, gender, year of diagnosis, ICD-O codes, affected organ, source of data, and divisional hospital of diagnosis. Ethnicity was the only category that was excluded due to internal MHMS regulations that prohibits the use of data pertaining to the ethnicity of a patient.

The age-standardized incidence and mortality rates were calculated using data on incidence and mortality (per 100 000 population). This methodology was chosen to make it possible to compare the results to those reported by the IARC for the period 2000 to 2025, which used the same standardization method.^12^ To calculate the mortality rate, the total number of cancer deaths over time was divided by the annual cancer incidence. The mortality percentage was then calculated by multiplying the resulting number by 1000. It should be noted that this mortality rate assumes that the deaths over time are the same patients who were diagnosed in the 8-year period.

The geospatial map was created using data from the MHMS on cancer incidence and death. The mortality data received included specific information on patient deaths within each subdivision, whereas the incidence data just included information on what the division of diagnosis was. This is due to the MCDC being used to explicitly inform the mortality data, which is gathered and prepared at a subdivisional level, allowing for granularity in mortality data as opposed to incidence data, which does not allow for this degree of granularity. Fiji is divided into administrative boundaries consisting of four major divisions, each with its own regional capital. There are 14 provinces across all four divisions, which are categorized as subdivisions.

The specific mortality rate (SMR) in the subdivisions was calculated in the current study by dividing the average number of breast cancer deaths from 2010 to 2018, in the average population of both males and females in the subdivisions using population census data collected in 2007 and multiplying the result by 100 000. The data was then transferred to a web mapping visualization software (Scribble Maps Pro) to understand the distribution of incidence and mortality of cancer throughout the different regions of Fiji. The methodology and format of this paper modeled a similar study that was conducted with cancer incidence and mortality data in Trinidad and Tobago.^13^

## Results

### Cancer Incidence and Mortality Among Men and Women

Table 1 presents the counts, percentages, and age-standardized incidence and mortality rates of cancer in both men and women, based on data available from 2010 to 2018. Over this period, a total of 12 433 cancer cases were reported, with 8457 cases in women and 3965 cases in men. A total of 5963 deaths were recorded during the same period, with 3753 being women and 2210 being men. The data reveal that women have higher cancer incidence rates than men, but men have a higher death rate, with a mortality rate of nearly 59%.

**Table 1.** Counts, Percentages, and Age-Standardized Incidence (A) and Mortality (B) Rates by 100 000 for Adult and Pediatric Cancers in the Fiji Islands 2010 to 2018.

(A) Incidence		Both sexes		Women		Men	
Cancer site	ICD-10 codes	Count	ASR-T	Count	ASR-F	Count	ASR-M
Bones and joints	C40, C41	141	3.3	74	2.3	67	4.3
Brain and other nervous system	C70-C72	178	4.1	74	2.1	104	6.0
Breast	C50	2231	36.1	2190	67.8	41	3.3
Digestive system	C15-26	2101	57.0	869	27.9	1232	91.0
Colon	C18	334	8.6	165	5.4	169	12.5
Liver	C22	370	16.0	128	6.4	242	26.9
Pancreas	C25	173	4.8	77	2.5	96	7.5
Rectum	C20	241	6.1	119	3.8	122	8.8
Stomach	C16	276	5.9	103	3.6	173	8.6
Other^a^	C15, 17, 19, 21, 23, 24, 26	707	13.9	277	6.6	430	22.6
Endocrine system	C73-C75	223	4.4	170	5.3	53	3.4
Eye and orbit	C69	30	0.8	13	0.4	17	1.1
Hematologic	C42, C77	606	14.3	271	8.1	335	20.8
Oral cavity and pharynx	C00-C14	308	7.9	134	4.1	174	12.1
Reproductive system	C51-C63	4129	76.6	3511	105.9	607	51.4
Penis	C60	18	0.6		0.0	18	1.3
Prostate	C61	500	19.6		0.0	500	44.5
Testis	C62	78	2.3		0.0	76	4.4
Other male genitals	C63	14	0.5		0.0	13	0.9
Cervix uteri	C53	2043	31.3	2043	62.3		0.0
Corpus uteri	C54	592	9.5	588	18.3		0.0
Ovary	C56	444	6.8	442	13.4		0.0
Vagina	C52	54	0.9	54	1.8		0.0
Vulva	C51	41	0.6	41	1.3		0.0
Other female genitals	C55, C58, C63	345	4.5	343	9.0		0.0
Respiratory system	C30-C34, C37-C39	594	15.9	262	8.4	332	24.6
Larynx	C32	51	1.6	8	0.3	43	3.3
Lung bronchus	C34	358	9.8	158	5.3	200	15.3
Other	C30, C31, 33, 37, 38	185	4.4	96	3.0	89	6.0
Skin (excluding basal and squamous)	C44	292	7.9	117	3.8	175	12.8
Soft tissue (including heart)	C47-C49	243	5.5	134	4.1	109	7.3
Urinary system	C64-C68	235	6.9	67	2.0	168	12.5
Ill-defined and unknown	C76, C80	1122	20.4	571	13.4	551	28.6
Total		12433		8457		3965	
(B) Mortality		Both sexes		Women		Men	
Cancer site	ICD-10 codes	Count	ASR-T	Count	ASR-F	Count	ASR-M
Bones and joints	C40, C41	75	1.8	40	1.3	35	2.1
Brain and other nervous system	C70-C72	75	1.8	30	0.9	45	2.8
Breast	C50	1111	18.1	1094	34.1	17	1.3
Digestive system	C15-26	1407	38.9	551	17.9	856	63.3
Colon	C18	154	4.3	67	2.3	87	6.5
Liver	C22	517	14.4	174	5.6	343	24.3
Pancreas	C25	145	3.9	66	2.1	79	6.0
Rectum	C20	116	3.0	60	2.0	56	4.1
Stomach	C16	172	4.8	64	2.1	108	7.9
Other^a^	C15, 17, 19, 21, 23, 24, 26	303	8.6	120	3.9	183	14.5
Endocrine system	C73-C75	107	2.3	79	2.5	28	1.8
Eye and orbit	C69	11	0.4	3	0.1	8	0.6
Hematologic	C42, C77	1	0.0		0.0	1	0.0
Oral cavity and pharynx	C00-C14	133	3.6	54	1.8	79	5.8
Reproductive system	C51-C63	1586	33.6	1226	37.6	360	32.5
Penis	C60	7	0.3		0.0	7	0.6
Prostate	C61	319	13.0	1	0.0	318	29.6
Testis	C62	32	1.0		0.0	32	2.0
Other male genitals	C63	2	0.0		0.0	2	0.0
Cervix uteri	C53	691	11.0	691	21.5		0.0
Corpus uteri	C54	162	2.8	161	5.1	1	0.0
Ovary	C56	303	4.8	303	9.3		0.0
Vagina	C52	9	0.1	9	0.3		0.0
Vulva	C51	8	0.1	8	0.3		0.0
Other female genitals	C55, C58, C63	53	0.8	53	1.4	0	0.1
Respiratory system	C30-C34, C37-C39	390	11.0	158	5.1	232	18.0
Larynx	C32	28	1.0	3	0.1	25	2.1
Lung bronchus	C34	3	8.9		4.6	3	14.0
Other	C30, C31, 33, 37, 38	359	1.1	155	0.4	204	1.9
Skin (excluding basal and squamous)	C44	28	0.8	13	0.4	15	1.1
Soft tissue (including heart)	C47-C49	65	1.5	31	0.9	34	2.3
Urinary system	C64-C68	98	2.9	30	1.0	68	5.1
Ill-defined and unknown	C76, C80	464	11.9	240	7.9	224	16.5
OTHER (D CODES)	D0-D48	412		204		208	
Total		5963		3753		2210	

a Anus, anal canal, bile tract, esophagus, gall bladder, gastrointestinal, rectosigmoid junction, and small intestine.

**Figure 1.**
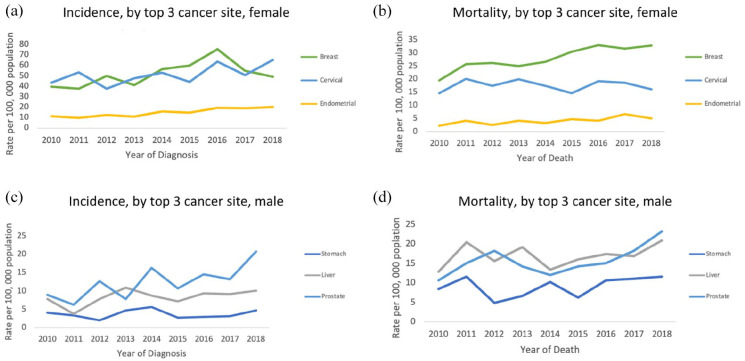
Trends in rates for the top three cancer sites by sex, Fiji Islands, 2010 to 2018. Rates are presented for top 3 cancer incidence in females (a), top 3 cancer mortality in females (b), top 3 cancer incidence in males (c) and top 3 cancer mortality in males (d).

Among women, breast cancer (67.8 per 100 000), cervical cancer (62.3 per 100 000), and endometrial cancer (18.4 per 100 000) were the top three cancer incidence rates, whereas prostate cancer (44.5 per 100 000), liver cancer (26.9 per 100 000), and lung cancer (15.3 per 100 000) were the top three cancer incidence rates among males. Similarly, breast cancer (34.1 per 100 000), cervical cancer (21.5 per 100 000), and ovarian cancer (9.3 per 100 000) had the highest mortality rates among women, whereas prostate cancer (29.6 per 100 000), liver cancer (24.3 per 100 000), and stomach cancer (7.9 per 100 000) had the highest mortality rates among males, as shown in Table 1 and Figure 1.

### Cancer Incidence and Mortality by Age

**Figure 2.**
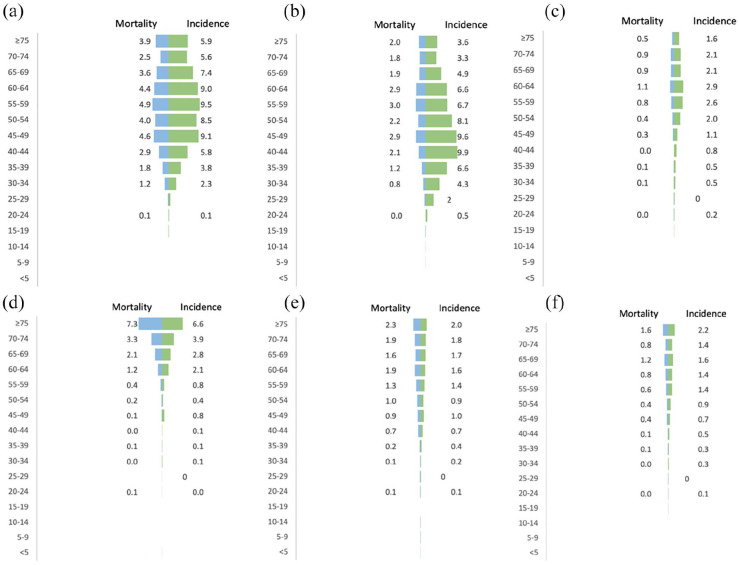
Age-standardized incidence and mortality rates for the leading cancer sites presented by sex and age groups. Cancers presented are breast cancer (a), cervical cancer (b), corpus uteri cancer (c), stomach cancer (d), liver cancer (e) and prostate cancer (f).

Figure 2 illustrates the age-standardized rates of cancer incidence and mortality categorized by age and gender. Breast and cervical cancers were found to be highest in women aged over 40 (Figure 2a-b), whereas corpus uteri cancer was observed to be more common in women aged 50 and above (Figure 2c). In men, stomach and liver cancers were more prevalent among those aged 50 and above (Figure 2d-e), whereas prostate cancer had a higher incidence rate in older men, with the greatest impact being observed among men aged 65 and above (Figure 2f).

### Cancer Incidence and Mortality by Geography

**Figure 3.**
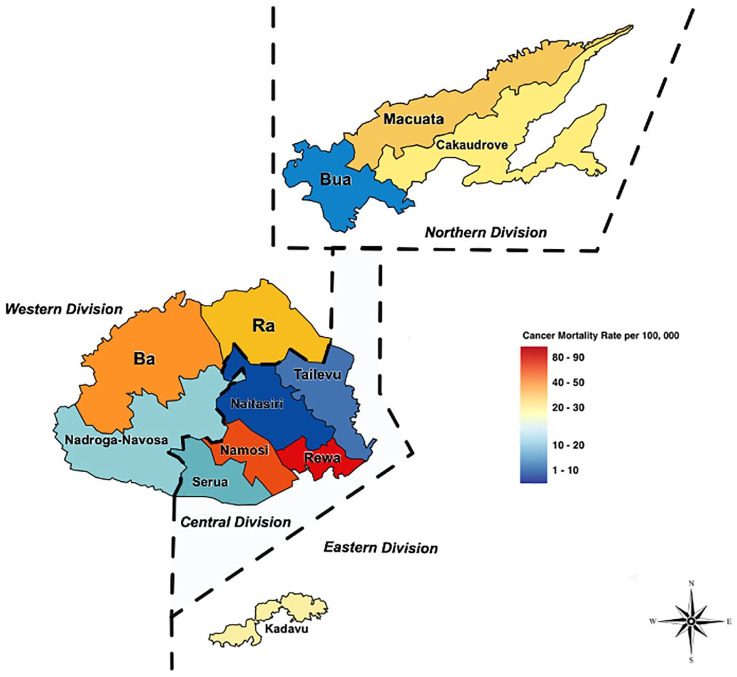
Geospatial maps of cancer mortality rates in Fiji, over a span of 8 years—2010 to 2018.

Figure 3 shows the cancer mortality rates across Fiji’s four divisions (Central, Western, Northern, and Eastern) from 2010 to 2018. The data account for all types of cancer and both male and female cases, further categorized by the respective subdivisions where the deaths occurred. Using a color-coded system, the map indicates the extent of mortality, with darker shades of red representing higher death rates and lighter shades indicating lower rates. Notably, the Rewa subdivision within the central division had the highest mortality rate of all regions, with moderately high rates observed in Macuata, Cakaudrove, Ba, and Ra. Meanwhile, Naitasiri and Tailevu exhibited the lowest mortality rates.

**Figure 4.**
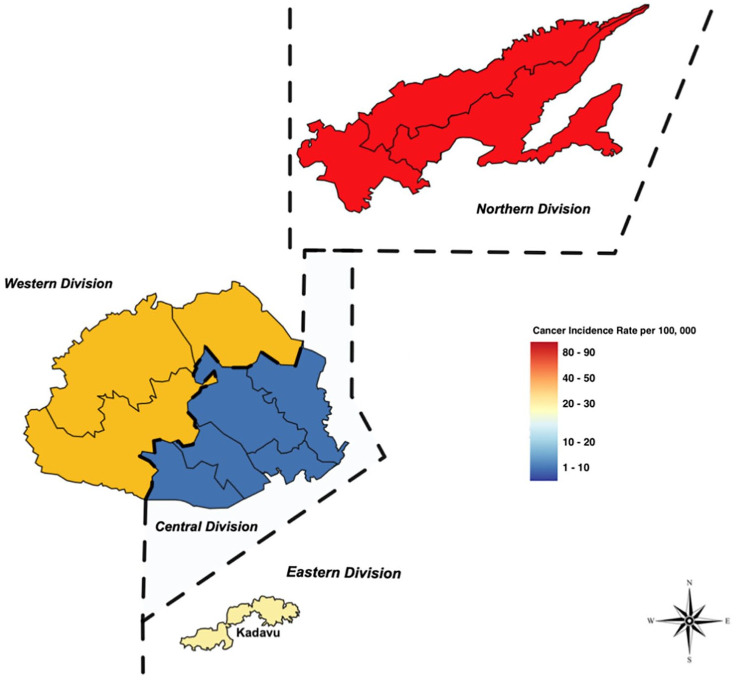
Geospatial maps of cancer incidence rates in Fiji, over a span of 8 years—2010 to 2018.

Figure 4, on the contrary, presents the cancer incidence rates in Fiji’s four divisions over the same period, incorporating both male and female data. Similarly color-coded, the figure reflects the levels of incidence, with darker shades of red suggesting higher rates and lighter shades suggesting lower rates. The Northern division exhibited the highest incidence rate, followed by the Western and Eastern divisions. By contrast, the Central division had the lowest rate.

## Discussion

The study provides an epidemiological analysis of cancer rates in Fiji, identifying prostate, liver, and stomach cancers as the most prevalent in men, and breast, cervical, and endometrial cancers in women. The study also identified that some regions in Fiji may disproportionately have greater mortality rates. Therefore, the study attempts to understand the socioeconomic and health care system factors associated with incidence and mortality rates in the Fiji Islands.

Non-communicable diseases (NCDs) constitute a staggering 80% of deaths in Fiji. Evidence shows that certain dietary, smoking, and drinking behaviors are strongly linked to several NCDs, including cancer, cardiovascular disease, and respiratory disorders.^8,14^ Alarmingly, only 15% of the population consumes sufficient fruits and vegetables, whereas 16.6% are daily smokers.^15^ The incidence of smoking-related cancers is particularly high among men, young adults, and iTaukei Fijian women (*iTaukeis* are descendants of the original inhabitants of Fiji). For instance, a study by the Ministry of Health & Medical Services (2011) found that although only 2.1% of Indo-Fijian women smoked, a staggering 23% of iTaukei women reported smoking. Furthermore, obesity rates have surged to 32.1%.^15^ Fiji also struggles with a pronounced prevalence of alcohol use disorder, with a striking 68.6% of Fijian adolescents engaging in excessive alcohol consumption.^16^

This study has found that among women, breast cancer, cervical cancer, and ovarian cancer are leading causes of mortality. This aligns with other Fijian reports, which echo these cancers as predominant among women.^10,17^ For example, the GLOBOCAN (2022) cancer fact sheet shows that the incidence of breast cancer is 38.9 per 100 000, which is similar to the 34.1 per 100 000 ASR that this article presents. This is higher than other neighboring Pacific Island countries’ mortality rates. For instance, in Papua New Guinea, the breast cancer mortality rate is 26.7 per 100 000.^18^ Breast cancer, in particular, imposes a significant health burden, representing the highest number of cancer cases nationwide in Fiji. Notably, there is a lack of established breast cancer policy and screening is often opportunistic, with mammography services primarily used for diagnostic purposes rather than screening. Furthermore, a 2018 report by the Fiji Women’s Rights Movement indicates that a considerable number of breast cancer cases are diagnosed at an advanced stage. Identifying the factors behind late-stage presentation is important in being able to reduce the high mortality rates associated with breast cancer.^19^

Cervical cancer had the second highest mortality rates; however, its mortality rate is lower than that for breast cancer. This could possibly be due to measures implemented such as a dedicated cervical cancer policy, widespread screening services, and the rollout of the HPV vaccine, which could have helped in early detection. In addition, there is a national screening policy for cervical cancer that dictates how screening services are provided across the four divisions, and there is an HPV vaccination program in place.^20^ On the contrary, prostate cancer stands as the most prevalent cancer among men in this study and by GLOBOCAN in 2020; however, despite the high incidence, there is a notable lack of literature on prostate cancer in Fiji detailing the reasons behind these rates^10^ noted that, due to the lack of a dedicated health care workforce and no routine prostate cancer screening services, there are lower levels of screening and higher rates of late-stage presentation in the male population.^21^

Using geospatial software, the study mapped cancer incidence and mortality across Fiji’s divisions. The Rewa subdivision reported high mortality, potentially affected by the centralized cancer care at Colonial Memorial Hospital, which draws patients from across towns and islands in Fiji, many of whom die in Rewa, inflating its mortality statistics. For example, researchers have also noted that a limitation with geographical information systems includes not being able to map incidence and mortality to a definable geographic location due to increased migration in countries.^22^ Therefore, to ensure an accurate representation of where the burden lies, there is a need for more granular data such as the residential address of the patients. However, it can be noted that other provinces, like Macuata, Ra, and Ba, also showed elevated mortality rates, which could be attributed to factors like health care access, awareness levels, and traditional beliefs that may hinder early cancer treatment. In addition, as per the 2007 census, the iTaukei population is the dominant ethnic group in these areas, indicating the need to explore what role ethnicity plays in treatment-seeking behavior and mortality rates of cancer.^17^ For example, research conducted in Australia has revealed that Indigenous cancer patients residing in rural and remote regions are associated with poorer survival rates compared with those living in urban regions and are 30% less likely to access cancer treatment facilities.^23^

It is important to note that the findings of this study have several limitations and should therefore be interpreted with caution. First, the quality of the cancer data overall was found to be low, with several entries missing critical information such as age, location of diagnosis or mortality, and type of cancer, as well as inaccurate gender allocations to gender-specific cancers. Second, the current data do not capture information on the stages of cancer at the time of diagnosis, nor does it collect information on the time between diagnosis and first recurrence. However, it is worth noting that information on cancer stage may exist in individual patient folders and pathological reports. This highlights the importance of expanding the registry’s capacity, which would be helpful in evaluating and monitoring screening and early detection programs. Third, there is a gap in the collection and documentation of treatment outcome data, and it is unclear whether the treatments and interventions currently in place are adequate to significantly reduce the burden of this disease in the country.

Urgent and comprehensive interventions are necessary to address the challenges described above and could potentially focus on improving dietary habits, reducing smoking rates, and promoting moderate alcohol use. Such actions are crucial in alleviating the burden of NCDs including cancer in Fiji, and consequently, cancer. It was noted that there is no established breast cancer policy, underscoring the critical need for strategic interventions to address this health challenge. In addition, given that this study provides a “snapshot” of Fiji’s cancer trends and burden, it underscores the need for improved cancer epidemiology and robust health monitoring systems. Finally, the high burden of cancer in Fiji is evident through the figures presented in this study, which make a strong case for improving cancer services across the Fiji Islands. This need is also echoed by the Impact Review Report conducted by the Programme of Action for Cancer Therapy (PACT) of the International Atomic Energy Agency (IAEA), the WHO, and the IARC, which suggested improvements in multiple areas of cancer control in Fiji, such as strengthening health system strategies for the control of cancers like breast, colorectal, and cervical cancer.^24^
